# Non‐zero‐sum neutrality test for the tropical rain forest community using long‐term between‐census data

**DOI:** 10.1002/ece3.8462

**Published:** 2022-01-17

**Authors:** Yayoi Takeuchi, Hisashi Ohtsuki, Hideki Innan

**Affiliations:** ^1^ Biodiversity Division National Institute for Environmental Studies Tsukuba Japan; ^2^ SOKENDAI (The Graduate University for Advanced Studies) Hayama Japan

**Keywords:** Barro Colorado Island, immigration, local birth rate per death, neutral model, number of new species, Pasoh

## Abstract

For community ecologists, “neutral or not?” is a fundamental question, and thus, rejecting neutrality is an important first step before investigating the deterministic processes underlying community dynamics. Hubbell's neutral model is an important contribution to the exploration of community dynamics, both technically and philosophically. However, the neutrality tests for this model are limited by a lack of statistical power, partly because the zero‐sum assumption of the model is unrealistic. In this study, we developed a neutrality test for local communities that implements non‐zero‐sum community dynamics and determines the number of new species (*N*_sp_) between observations. For the non‐zero‐sum neutrality test, the model distributed the expected *N*_sp_, as calculated by extensive simulations, which allowed us to investigate the neutrality of the observed community by comparing the observed *N*_sp_ with distributions of the expected *N*_sp_ derived from the simulations. For this comparison, we developed a new “non‐zero‐sum *N*_sp_ test,” which we validated by running multiple neutral simulations using different parameter settings. We found that the non‐zero‐sum *N*_sp_ test rejected neutrality at a near‐significance level, which justified the validity of our approach. For an empirical test, the non‐zero‐sum *N*_sp_ test was applied to real tropical tree communities in Panama and Malaysia. The non‐zero‐sum *N*_sp_ test rejected neutrality in both communities when the observation interval was long and *N*_sp_ was large. Hence, the non‐zero‐sum *N*_sp_ test is an effective way to examine neutrality and has reasonable statistical power to reject the neutral model, especially when the observed *N*_sp_ is large. This unique and simple approach is statistically powerful, even though it only employs two temporal sequences of community data. Thus, this test can be easily applied to existing datasets. In addition, application of the test will provide significant benefits for detecting changing biodiversity under climate change and anthropogenic disturbance.

## Graphical abstract

In this study, we developed a new neutrality test, so‐called “non‐zero‐sum *N*_sp_ test,” for local communities that implements non‐zero‐sum community dynamics and examines neutrality based on the number of new species between censuses. For an empirical test, we applied the test to real tropical tree communities in Panama and Malaysia, and it rejected neutrality in both communities.

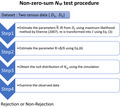

## 1 INTRODUCTION

The concept of “neutrality,” which indicates the principles of demographically equivalent individuals, has been applied in a wide range of fields, including ecology, genetics, economics, and sociology. This concept has made significant contributions to progress in every field, both philosophically and technically. For example, in community ecology, intensive arguments over neutrality were ignited by when he introduced his explicit neutral model (Hubbell 2001) 20 years ago. Before Hubbell's neutral model appeared, several pure statistical (neutral) models, such as broken‐stick, log‐normal, logarithmic, and geometric series, were commonly used as an alternative or null model to examine the deterministic processes that shaped biological community structure. Generally, deterministic models provided better explanatory power than these statistical neutral models. However, Hubbell's neutral model has exhibited a good fit to several natural communities [e.g., tropical forests (Etienne, 2005; Volkov et al., 2005), fishes (Etienne & Olff, 2005), and birds (He, 2005)]. These discrepancies aroused ecologists' interest and Hubbell's neutral model, as well as alternative deterministic models (Chesson, 2000; Tilman, 1982; Tokeshi, 1990, 1992) have been intensively studied, theoretically and empirically.

One of the significant technical merits of Hubbell's neutral model is its simple form of community dynamics, with few parameters and simple assumptions; it enables us to provide plausible predictions of spatiotemporal patterns in species diversity. Thus, Hubbell's neutral model is considered an “efficient theory,” which entails few assumptions and free parameters under the “first principle” (Marquet et al., 2014). However, despite long‐term debate, there is currently no consensus on the role of Hubbell's neutral model. This lack of clarity is partly because the application of Hubbell's neutral model covers a wide range of purposes and interpretations (Adler et al., 2007; Gotelli & McGill, 2006), which were derived from different philosophical backgrounds (Munoz & Huneman, 2016; Wennekes et al., 2012). A recent study by Leroi et al. (2020) highlighted the analogy of the history of neutral models in various fields; “when first introduced, neutral theory has always been accompanied by claims that the world (or some aspect of it) really is neutral. Much quarreling ensues, many tests—weak at first, but incrementally increasing in power—are offered, a truce is forged as the theory turns into a null model against which to infer selection or else a useful modelling tool.” In this sense, community ecology is on its way to the last phase; we are still struggling to develop the framework to use Hubbell's neutral model as a null model, or even a neutral test for this model, even though its significant role as a null model (Adler et al., 2007; Alonso et al., 2006) over a deterministic model has often been emphasized. In particular, for community ecologists, “neutral or not?” is a fundamental question when investigating the ecological processes underlying community dynamics. Examining neutrality is a basic starting point because neutrality must be rejected before one explores alternative models to detect the essential (non‐neutral) processes that form community structure and composition. However, because of the absence of effective neutrality tests (Takeuchi & Innan, 2015), community ecology has not reached the last phase yet. Thus, it is essential to develop a powerful neutrality test for Hubbell's neutral model, which would also contribute to the next step, that is, developing the framework to use this neutral model as a null model or exploring alternative deterministic models.

**FIGURE 1:**
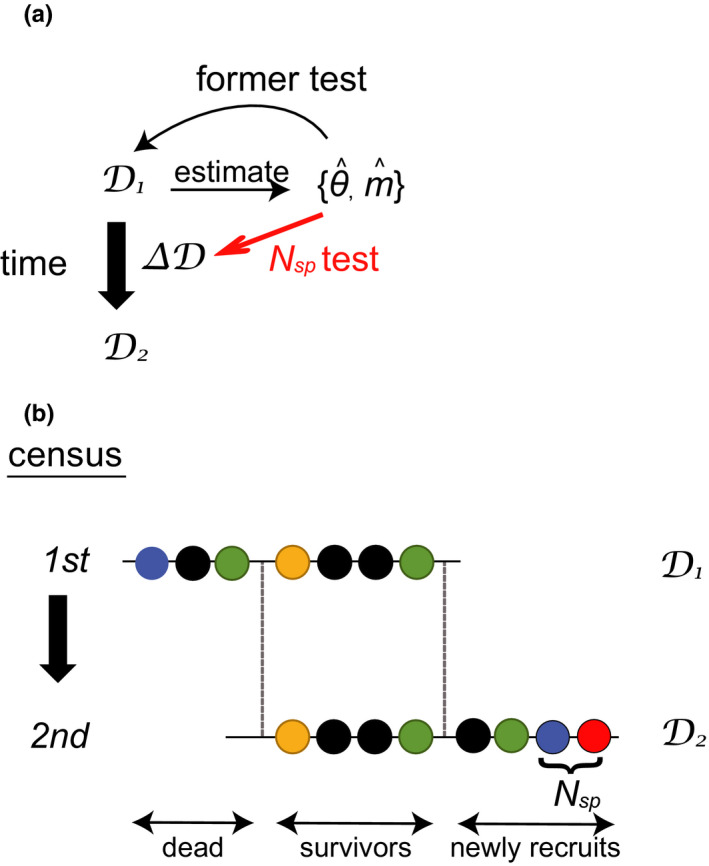
(a) Commonly used SAD‐based neutrality tests are based on a single census, which results in good model fit due to model flexibility. Our new test is based on two censuses, which partially overcome this problem. (b) Schematic overviews of non‐zero‐sum dynamics in a local community, assuming that all individuals in a local community are distinguished and their fates are recorded. At *D*_1_, there are seven individuals from four species. Between the first and second census, three individuals died and four individuals from three species survive. Four individuals of four different species are newly recruited through immigration or local birth. Among these four, two individuals (blue and red) are defined as new species. Note that according to our definition of “new”, explained in the main text, the blue individual in the second census is counted as “new” even though a blue individual was observed in the first census, because blue individuals were not observed among the survivors from the first census

Hubbell's neutral model applies three important assumptions of “neutrality.” The first assumption involves demographic equivalence among species. All individuals are symmetrical, either ecologically or functionally, with respect to demography; in other words, the community has no interindividual differences regarding the level of fitness. The second assumption involves zero‐sum dynamics with ecological drift. The size of the community in Hubbell's neutral model remains constant over time, and the composition of the local community is determined by stochastic extinction, local birth, and dispersal from the nested metacommunity (i.e., ecological drift). The third assumption involves random speciation. Species in a metacommunity are derived from a point mutation process. The two parameters that concisely summarize the model are the fundamental biodiversity number, *θ*, which determines the species diversity of the metacommunity, and the immigration rate, *m*, which describes the migration from the metacommunity to the local community. Fitting Hubbell's neutral model has been criticized because the flexibility of the two parameters of the model can result in a “false good fit” of the species abundance distribution (SAD) (Adler et al., 2007; Bell, 2005; Chave, 2004; Chisholm & Pacala, 2010; McGill et al., 2007; Takeuchi & Innan, 2015). This can be a critical problem when neutrality tests are applied to SAD data obtained from one community at one point in time, which was the case for false good fits in almost all previous studies. In such cases, the values of *θ* and *m* were estimated from the observed SAD of the focal local community alone, and then, its neutrality was tested using the same data used to estimate the parameters (see Figure 1a), thereby resulting in an apparently good fit to the neutral model, with a reduced capability of rejecting neutrality (Takeuchi & Innan, 2015). Additional data, such as temporal sequence data for SAD, can dramatically improve the statistical power because those data can compensate for the uncertainty of one‐time data used for parameter estimation and/or can provide independent information on the focal community dynamics (Kalyuzhny et al., 2014, 2015; Washburne et al., 2016).

The second and third assumptions of the above‐described neutrality model have been criticized for being unrealistic and naive (Rosindell et al., 2011, 2012). These “auxiliary assumptions” of Hubbell's neutral model include constant local community size *J* (zero‐sum dynamics) and speciation by point mutation, which are unrelated to the neutrality of community dynamics but would cause the model to fail to reject neutrality when appropriate (Rosindell et al., 2011). Particularly, the zero‐sum assumption applies the dynamics that as one individual dies, it is immediately replaced with another individual through birth within the local community or immigration from the metacommunity. This assumption is obviously inconsistent with real communities because the zero‐sum feature of the local community size is not guaranteed in every instance. A non‐zero‐sum community results in an inconstant *m* over time, which also violates the assumptions of Hubbell's neutral model (Etienne et al., 2007).

For robust “neutrality” testing, it is desirable to overcome these limitations of producing unrealistic models. As for overfitting of the model, we could apply additional information that is independent of the information used in parameter estimation. For example, temporal sequences of community composition have been used to examine deviations from the expectations set by Hubbell's neutral model. The population abundance per species over time (Chisholm et al., 2014; Jabot & Lohier, 2016; Kalyuzhny et al., 2015), covariance structure of fluctuations in relative population abundance (Washburne et al., 2016), and species composition over time (Kalyuzhny et al., 2015) have been considered, although those models require an additional parameter to explain temporal fluctuations in the community, that is, temporal autocorrelation, time‐dependent fitness fluctuation, or species‐specific birth rate, which may differ from the neutral assumption. To overcome the problems associated with the zero‐sum assumption, we could simply develop a non‐zero‐sum model; in fact, such models were developed in previous studies (Etienne et al., 2007; Haegeman & Etienne, 2008; O'Dwyer & Cornell, 2018). The non‐zero‐sum model is a straightforward extension of the original Hubbell's neutral model that adopts the zero‐sum assumption.

Here, we propose a simple new neutrality test that uses community composition (SAD) and dynamic data obtained from a single community at two different time points and allows for a non‐zero‐sum community size. For each pair of census data (*D*_1_ and *D*_2_, Figure 1a), differences in species composition (Δ*D*) were calculated. In particular, we focused on the number of new species in the second census, denoted as *N*_sp_. Here, *N*_sp_ is defined as the number of species that were observed among the recruited individuals after the first but before the second census, yet were not seen among the individuals that survived from the first census (Figure 1b). We incorporated datasets that record individual demographical history, such as forest plot monitoring data.

This concept allowed us to develop a new *N*_sp_‐based statistical test of neutrality, comparing census data at two different time points. Since we applied non‐zero‐sum dynamics, this test was called a “non‐zero‐sum *N*_sp_ test.” To examine the test, we estimated the parameters as follows. First, we used a fundamental biodiversity number, *θ*, which represents the rate of speciation in the metacommunity. Second, we used the rate of immigration to the local community per local birth per individual, *I*. Here, a constant migration rate, *m*, cannot be used because as the local community grows (or shrinks), the relative impact of immigration changes (see the relationship between *I* and *m* shown below). Third, we used *R*, which is the intrinsic growth rate of the local community, based on observed census data (*D_t_*). Contrary to the zero‐sum model, the non‐zero‐sum model requires estimation of the additional parameter, *R*. Moreover, because of the absence of a formula that gives the distribution of *N*_sp_ under the non‐zero‐sum model, extensive simulations are required to estimate the *N*_sp_ distribution. Our test was designed in such a way that the values estimated using the first census (*D*_1_) were used to test the difference between the two censuses (Δ*D*), as illustrated in Figure 1a. This testing design provides a partial solution to the problem of overfitting to Hubbell's neutral model. Additionally, we considered a non‐zero‐sum community, which removes the “auxiliary assumption” of zero‐sum dynamics. The performance of the non‐zero‐sum *N*_sp_ test was validated by simulating communities under the non‐zero‐sum neutral model. We found that the non‐zero‐sum *N*_sp_ test performed appropriately. As a real‐world test, the non‐zero‐sum *N*_sp_ test was applied to datasets from species‐diverse tropical tree communities in Panama and Malaysia. These datasets are long‐term monitoring plots that are commonly used for model testing in community ecology and are, therefore, appropriate data sources for our test. Based on time census data, the non‐zero‐sum *N*_sp_ test rejected neutrality in both communities, which was contrary to former neutrality tests that applied data from a single point in time.

Our testing design is unique and simple in that it employs only two temporal sequences of community data and focuses on *N*_sp_ between the two observations. Observations of community dynamics are conducted in many types of ecosystems and the resulting data are also commonly available; thus, performing our test using existing data would not be difficult. The test developed here has substantial power to reject demographic neutrality in community dynamics. The application of this test will provide significant benefits for community ecology, especially for detecting changing biodiversity under climate change and anthropogenic disturbance.

## 2 METHODS

### 2.1 Non‐zero‐sum neutral model

We applied a non‐zero‐sum model of neutral community developed by Etienne et al. (2007). The model constructs two communities, which are a local community nested within a metacommunity, the latter of which is assumed to be a large community where speciation occurs. We mainly focused on the species composition of the local community. Suppose that the local community size is J. Our stochastic process comprises three different types of random events in the local community, which are birth, death, and immigration, and each rate is βJ, δJ, and λ, respectively. If a birth event occurs, a random individual in the local community reproduces, which changes the local community size from J to J+1. If a death event occurs, a random individual in the local community dies, which changes the local community size from J to J‐1. If immigration occurs, a random individual migrates from the metacommunity, which changes the local community size from J to J+1. A fundamental biodiversity number is assumed to be θ, which will be used later.

Under such assumptions, the first census data of the entire local community are denoted as $D_{1}.$ Assuming that the local community size of the first census is $J_{1}$, Haegeman and Etienne (2008) showed that the probability distribution of the composition of this local community matches the one calculated by assuming that a zero‐sum model of the local community size is fixed to $J_{1}$, for which the explicit likelihood function was described by Etienne and Alonso (2005) and Etienne (2007). In the zero‐sum model by Hubbell (2001), each death in the local community is compensated by an immigration or a local birth with probabilities m and 1‐m, respectively. In terms of our non‐zero‐sum model parameters, the probability of immigration m was the proportion of immigrants among all recruited individuals, thus rewritten as $m=\lambda /\left\{\lambda +\beta \left(J_{1}‐1\right)\right\}$, so we have the following equation,

(1) $$m=\frac{I}{I+J_{1}‐1}\;\text{or equivalently},\;I=\left(J_{1}‐1\right)\frac{m}{1‐m},$$

where we introduced the parameter I:=λ/β [see Etienne et al. (2007)], which is immigration per local birth by a single individual. In the non‐zero‐sum community model, this parameter controls the community size.

After the first census, there are birth, death, and immigration events in the local community during a certain time interval, after which a second census is conducted. Data from the second census are denoted by $D_{2},$ and the local community size at this second census is annotated as $J_{2}.$ The $J_{2}$ individuals are categorized as survivors and new recruits. Survivors refer to those who survived between the two censuses, and the number is represented as $J_{1}‐d$. Here, d represents the number of individuals that died between the censuses (“*d*” for “dead”). Recruits are those that were absent in the first census but present in the second census. Recruits may have been produced from the offspring of individuals that were counted in the first census, migrants between the two censuses, or descendants of those migrants. Here, the number is represented by r (“*r*” for “new recruits”). Hence, we have the following equation:

(2) $$J_{2}=\left(J_{1}‐d\right)+r.$$

We know $D_{1}$ and $D_{2}$ (and, therefore, $J_{1}$ and $J_{2}$) on the basis of actual census data. It is also assumed that we know which individuals in the second census are survivors from the first; therefore, we know d and r. With these census data, we invent and propose a new neutrality test for examining the neutrality of community processes.

### 2.2 Procedure of non‐zero‐sum neutrality test (Figure 2)

#### 2.2.1 Step 1

For the estimation of two parameters, θ and I, in our model, we use the previous zero‐sum‐community model and apply the maximum‐likelihood estimation (MLE) method developed by Etienne (2007), which is applied to our first census data, $D_{1}$. The MLEs, $\hat{\theta }$, and $\hat{m}$ were obtained using the PARI/gp program (http://pari.math.u‐bordeaux.fr/), which is a widely used free computer algebra system. To estimate $\hat{\theta }$ and $\hat{m}$ from community data, we used the algorithm provided by Etienne (2007). Several maxima may be seen in the MLE approach. For estimating I, we use Equation (1) and obtain

(3) $$\hat{I}=\left(J_{1}‐1\right)\frac{\hat{m}}{1‐\hat{m}}.$$

#### 2.2.2 Step 2

Next, as we consider a non‐zero‐sum process, we estimate R=β/δ, which is the intrinsic growth rate of the local community, by relying on a deterministic approximation. Consider a continuous time model and suppose that the size of a group (called the “survivor group”) at time τ is given by $X_{\tau }$. This group starts with $X_{0}=J_{1}$ (which is the first census) and monotonically declines at a rate δ as

(4) $$\frac{dX}{d\tau }=‐\delta X.$$

and ends at time $\tau =\tau^{*}$ (the second census) in the formula $X_{\tau^{*}}=J_{1}‐d.$ The size of another group (called the “newly recruited”) at time τ is given by $Y_{\tau }$. It starts with $Y_{0}=0$ and changes according to

(5) $$\frac{dY}{d\tau }=\beta \left(X+Y\right)‐\delta Y+\lambda .$$

On the right‐hand side of Equation (5), the term $\beta \left(X+Y\right)$ represents local birth, δY represents local death among newly recruited individuals, and λ represents immigration. Solving Equations (4) and (5) by using boundary conditions that are consistent with the observed census data, $X_{0}=J_{1}$, $X_{\tau^{*}}=J_{1}‐d$, $Y_{0}=0$, and $Y_{\tau^{*}}=r$, while using $\hat{I}=\lambda /\beta$ and R=β/δ, the following equation is obtained with respect to R:

(6) $$\left(J_{1}+\frac{\hat{I}}{1‐\frac{1}{R}}\right)\left\{{\left(\frac{J_{1}‐d}{J_{1}}\right)}^{1‐R}‐1\right\}=r‐d.$$

The only unknown parameter in Equation (6) is *R*. The solution to Equation (6) with respect to *R* is used as an estimate of R, which is written as $\hat{R}$.

#### 2.2.3 Step 3 (null distribution of $N_{\text{sp}}$)

The absolute values of the three demographic parameters, β, δ, and λ, are relevant only to the speed of change, and their relative values are required. Thus, we can set β=1 without a loss of generality. Given the three parameter estimates obtained in *Steps 1* and *2*, $\hat{\theta }$, $\hat{I}=\left(\hat{\lambda /\beta }\right)$, and $\hat{R}=\left(\hat{\beta /\delta }\right)$, parameters necessary to run our simulations are set as

(7) $$\beta =1,\;\delta =1/\hat{R},\;\lambda =\hat{I},\;\text{and}\;\theta =\hat{\theta }.$$

To obtain the distribution of the number of new species $N_{\text{sp}}$ under the null hypothesis that the community is neutral, we created a neutral local community of size $J_{1}$ according to Etienne's algorithm (2005), with $\theta =\hat{\theta }$ and $m=\hat{I}/\left(\hat{I}+J_{1}‐1\right)$, while keeping track of the origin of each individual that immigrated from a metacommunity. The local community was then updated according to the parameters in Equation (7) and the procedure explained in *Step 1*, while taking note of their ancestral lineages. Whether individuals of different origin in migration can be of the same species depends on the magnitude of $\theta =\hat{\theta }.$ When d out of $J_{1}$ (where $J_{1}$ represents the number of individuals who were present in the first census) die, we check the current local community size. If the size is exactly equal to $J_{2}$, we adopt this simulation and record the number of new species, $N_{\text{sp}}$. If the local community size is not exactly equal to $J_{2}$, this simulation is abandoned. This rejection sampling procedure is repeated until there are a predetermined number of samples of $N_{\text{sp}}$, which is used as a null distribution of $N_{\text{sp}}$ (see Supplementary code). Here, we determine the percentile of the distribution (e.g., 2.5%, 97.5%) for a significance level of α=0.05 and evaluate whether the observed *N*_sp_ is outside of the range. When *N*_sp_ is outside the range, the non‐zero‐sum neutral model is rejected (i.e., two‐tailed test). Alternatively, there might be situations where a one‐tailed test is applicable. For example, in a tropical forest community, disturbance might increase *N*_sp_ because of frequent species turnover (Laurance et al., 2006). For this case, a one‐tailed (upper tail) test can achieve better performance.

**FIGURE 2:**
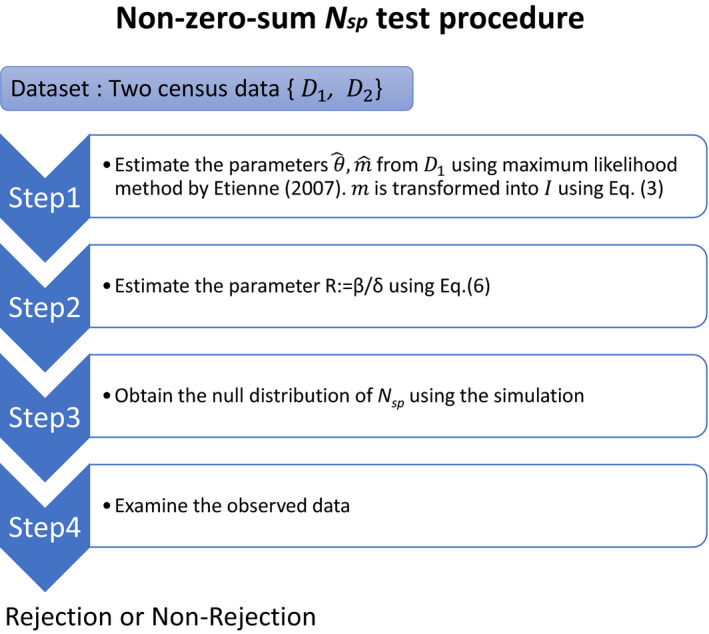
Schematic overview of our non‐zero‐sum *N*_sp_ test

Among the steps in Figure 2, the parameter estimations and transformation in *Step 1* rely on the conventional approach developed by Etienne (2007) and similar to the parameters used in Hubbell's zero‐sum neutral model. Other parameters and the $N_{\text{sp}}$ distribution in *Steps 2* and *3* were specific to the non‐zero‐sum model, introduced here.

### 2.3 Validation

To confirm the statistical power of our non‐zero‐sum *N*_sp_ test, we performed an extensive neutral simulation experiment against various parameter sets. For this validation, the parameters $\left(J_{1}^{*}.\theta^{*},I^{*},R^{*}\right)$were set in advance and used to generate a simulated community several times under the non‐zero‐sum neutral model. First, we produced the first census of the simulated community for the parameters $\left(J_{1}^{*}.\theta^{*},I^{*},R^{*}\right)$ by using Etienne's algorithm [Appendix S2 of (Etienne, 2005)], where the migration rate m was calculated using Equation (1). Next, we ran a neutral simulation as follows. A single simulation included y events and each event was either a local death, local birth, or immigration, the occurrence of which depended on the prespecified parameters $\left(I^{*},R^{*}\right)$ and the current local community size. We obtained a second‐census sample of the simulated community at the end of the *y* events. Then, *N*_sp_ was calculated by comparing simulated communities from the first and second censuses and used to validate our test. If our neutrality test was valid, we expected the proportion of simulations where neutrality was rejected to be nearly equal to the predetermined significance level of the test. Conversely, if the proportion of rejection was higher than the predetermined significance level, our new test produced several false positives. We used six different parameter sets (Table 1), which were close to the estimated parameters for tropical forest communities in Barro Colorado Island (BCI) and Pasoh (Etienne, 2005). In BCI, parameter estimation using the MLE yielded two optima; thus, both sets were considered. Here, 1000 simulations were obtained for each parameter set. For each simulated community, we recorded *D*_1_ and *D*_2_ and then examined the non‐zero‐sum *N*_sp_ test using the procedure described above (Figure 2). For the test, we applied a two‐tailed test and a one‐tailed test (upper tail) using the *N*_sp_ distribution. Here, a one‐tailed test (upper tail) was appropriate for a tropical forest community because the observed *N*_sp_ values were mostly larger than expected under the non‐zero‐sum neutral model (see Table 2).

**TABLE 1:** Simulation parameters and the validation results for the non‐zero‐sum *N_sp_* test

Parameter set #	*θ*	*I*	*J*_1_	Number of events (y)	Number of rejections	
					Two‐tailed	One‐tailed (upper)
1	50	2222	20,000	4500	25	43
2	250	60	20,000	4500	18	31
3	200	3333	30,000	5500	33	35
4	50	2222	20,000	18,000	45	38
5	250	60	20,000	18,000	63	71
6	200	3333	30,000	20,000	46	34

Note

Number of rejections indicates the number of simulations that rejected neutrality among 1000 simulation runs. Assumed *R* = 0.9.

**TABLE 2:** Summary of monitoring, the estimated parameters, and results of the non‐zero‐sum *N*_sp_ test for BCI and Pasoh

Site	Year1	Year2	Interval (year)	*J*_1_	*d*	*r*	*J*_2_	*N*_sp_	*θ*	*m*	*I*	*R*	*N*_sp_ test				
													Two‐tailed			One‐tailed (upper)	
BCI	1982	1985	3	20,882	1840	2036	21,078	5	47.28	0.15	3684.9	0.94	‐	6 (8)	NS	6 (7)	NS
BCI	1985	1990	5	21,078	2124	2890	21,844	4	47.87	0.15	3719.5	1.27	‐	8 (9)	NS	7 (9)	NS
BCI	1990	1995	5	21,844	1939	2254	22,159	2	48.58	0.1	2427.0	1.04	‐	6 (7)	NS	6 (7)	NS
BCI	1995	2000	5	22,159	2279	1998	21,878	4	48.28	0.11	2738.6	0.78	‐	6 (7)	NS	5 (7)	NS
BCI	2000	2005	5	21,878	2474	2173	21,577	3	47.81	0.11	2703.9	0.79	‐	6 (8)	NS	6 (7)	NS
BCI	2005	2010	5	21,577	2357	2350	21,570	2	46.89	0.12	2942.2	0.88	‐	7 (8)	NS	6 (7)	NS
BCI	1985	2005	20	21,078	7628	8127	21,577	19	47.87	0.15	3719.5	0.90	4 (2)	19 (21)	*	17 (20)	*
BCI	1990	2010	20	21,844	7995	7721	21,570	13	48.58	0.10	2427.0	0.87	3 (1)	16 (19)	NS	15 (18)	NS
BCI	1982	2005	23	20,882	8842	9537	21,577	27	47.28	0.15	3684.9	0.90	6 (4)	22 (24)	**	21 (23)	**
BCI	1985	2010	25	21,078	9212	9704	21,570	21	47.87	0.15	3719.5	0.89	6 (4)	23 (26)	NS	21 (24)	*
BCI	1982	2010	28	20,882	10,262	10,950	21,570	29	47.28	0.15	3684.9	0.89	8 (6)	26 (29)	**	24 (28)	NS
Pasoh	1985	1990	5	26,551	891	2283	27,943	4	191.24	0.09	2625.8	2.28	1 (0)	12 (14)	NS	11 (13)	NS
Pasoh	1990	1995	5	27,943	2274	3996	29,665	16	192.32	0.08	2429.7	1.57	3 (2)	17 (20)	NS	16 (19)	*
Pasoh	1995	2000	5	29,665	2707	2178	29,136	8	194.8	0.07	2232.8	0.75	0 (0)	11 (13)	NS	10 (12)	NS
Pasoh	2000	2005	5	29,136	2980	2394	28,550	12	195.86	0.07	2193.0	0.75	1 (0)	12 (14)	*	11 (13)	*
Pasoh	2005	2010	5	28,550	3225	3210	28,535	9	195.61	0.08	2482.5	0.92	2 (1)	16 (18)	NS	14 (17)	NS
Pasoh	1985	2005	20	26,551	7716	9715	28,550	45	191.24	0.09	2625.8	1.11	19 (16)	43 (47)	*	41 (45)	**
Pasoh	1990	2010	20	27,943	9764	10,356	28,535	46	192.32	0.08	2429.7	0.97	21 (18)	45 (49)	*	43 (47)	*
Pasoh	1985	2010	25	26,551	10,047	12,031	28,535	54	191.24	0.09	2625.8	1.05	28 (24)	54 (60)	*	52 (57)	*

Note

Significant levels of the non‐zero‐sum *N*_sp_ test.

NS: not significant.

* *p* < .05; ** *p* < .01.

### 2.4 Application of the non‐zero‐sum *N*_sp_ test to tropical forest communities

The non‐zero‐sum *N*_sp_ test was applied to datasets from tropical forest tree communities in 50‐ha plots in BCI, Panama, and Pasoh Forest Reserve, Malaysia. In both plots, trees ≥1 cm dbh were tagged, mapped, and identified as species and censused every 3–5 years. Hence, we accounted for the death, survival, and recruitment of all trees recorded in the census. Data for BCI were obtained from 1982 to 2005 (six censuses), and data from the Pasoh plot were obtained between 1985 and 2005 (five censuses; Table 2). The non‐zero‐sum *N*_sp_ test was applied to all pairwise comparisons of census data recorded for each plot.

## 3 RESULTS

### 3.1 Validation of the non‐zero‐sum *N*_sp_ test

**FIGURE 3:**
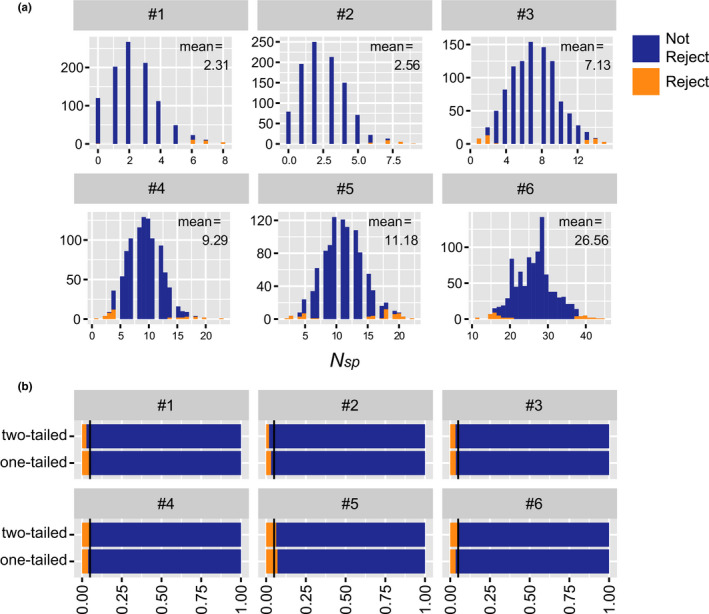
Summary of validation. (a) Histogram of *N*_sp_. Cases that rejected the non‐zero‐sum neutral model (*α* = 0.05) are shown in orange and the others are shown in blue for each non‐zero‐sum *N*_sp_ test (two‐tailed). (b) The proportion of simulations that rejected, or not, the neutral model (*α* = 0.05) is shown for each non‐zero‐sum *N*_sp_ test [two‐tailed, one‐tailed (upper)]. The vertical black lines indicate 5% of the proportion of the results of 1000 simulations in each parameter set, #1 to #6

We applied the non‐zero‐sum *N*_sp_ test to the simulated data under neutral assumptions for different parameter sets. The histogram of *N*_ap_ derived from the simulations is summarized in Figure 3a (see also Figure S1 for the other parameters). For 1000 simulated communities with a significance level of α=0.05, neutrality was rejected in less than 50 cases by our new non‐zero‐sum *N*_sp_ test (*p* < .05; Figure 3b), except for parameter set #5. The statistical power to reject the non‐zero‐sum neutral model was conservative. Moreover, for the most conservative case (i.e., the rejection rate was ~2% and was a two‐tailed test with parameter set #2), the observed *N*_sp_ was small when *J* and the number of immigrants were small; thus, rejection only occurred at the upper end of the *N*_sp_ distribution (Figure 3a). In other words, a smaller number of the observed *N*_sp_ was insufficient to reject neutrality. A fair test can be performed only when *J* is large or when longer‐term monitoring enables the observation of more birth, death, and immigration events.

### 3.2 Neutrality of tropical forest communities

We applied the non‐zero‐sum *N*_sp_ test to the two tropical forest communities, and Table 2 summarizes a subset of the results. According to the steps of the test (Figure 2), we first estimated the two parameters by Etienne's MLE method from the first census. For BCI, the estimated parameters were $\hat{\theta }$ ~ 48 and $\hat{m}$ ~ 0.13; and for Pasoh, the estimated parameters were $\hat{\theta }$ ~ 192 and $\hat{m}$ ~ 0.08. The estimated parameters between each census were nearly identical (Table 2). Generally, the observed *N*_sp_ was larger than expected under neutrality, except for one case in each forest. As was suggested from the results of the validation, the statistical power to reject the non‐zero‐sum neutral model depended on either *N*_sp_ or the time interval; with a larger *N*_sp_, the statistical power increased, which indicated that longer‐term observation with a sufficient size of *N*_sp_ is necessary to obtain meaningful results. The rejection of neutrality in two tropical forests indicates the importance of maintaining non‐neutral processes in these communities, which is consistent with concepts proposed by previous studies (Adler et al., 2007; Bell, 2005; Chave, 2004; Chisholm & Pacala, 2010; Jabot & Chave, 2011; Takeuchi & Innan, 2015).

## 4 DISCUSSION

Our non‐zero‐sum neutrality test using *N*_sp_ highlights two notable points when comparing our present model to previous neutrality tests. First, this test partially overcomes the problems of overfitting parameters by applying a parameter, that is, *N*_sp_ (Figure 1a). As noted by the previous study by Takeuchi and Innan (2015), commonly used SAD tests rely on two important parameters (*θ* and *m*), which must be estimated from the observed data. This loop (as Figure 1a shows) overfits the neutral model to the observed data, causing a marked reduction in the statistical power of the null neutral model. Although our non‐zero‐sum *N*_sp_ test still depends on the estimated *θ* and *m*, the values do not have to be estimated from the observed *N*_sp_; in fact, we recommend that the parameters are estimated from the first census data (*D*_1_ in Figure 1a). We believe that this could be one reason why the non‐zero‐sum *N*_sp_ test is successful. Using an independent parameter that is not used for parameter estimation allows us to perform more robust testing for community dynamics generally. Second, our model overcomes an auxiliary assumption, that is, zero‐sum dynamics. In previous neutrality tests, a zero‐sum community was typically assumed. However, examination of the SAD is unnecessary, as the non‐zero‐sum dynamics model predicts the same SAD (Etienne et al., 2007). Conversely, our test uses the *N*_sp_, which is produced by community dynamics (death, birth, and immigration) over time. Community size varies with events/time, and our model incorporates this stochasticity.

Our non‐zero‐sum model employed two key parameters *I* (immigration per local birth by a single individual) and *R* (a local birth rate per death), which would suggest the relative importance of immigration and local births. The parameter, *R*, indicates the intrinsic growth rate of the local community and reveals the regeneration process. In the case of Pasoh, mass flowering events occur only once per several years, and massive community size expansion therefore happens as a result of such events because of the increased number of local births. The difference in *R* values would reflect the event; for example, the highest value of *R* in Pasoh was in 1985–1990, which resulted from a mass flowering event in 1981 (Appanah, 1985). On the other hand, the parameter, *I*, reveals the relative importance of immigration over local births; for example, the highest value of *I* in BCI was observed in 1985, just after an extremely severe drought in early 1983 associated with a strong El Niño event. As this drought caused tree death, community size expansion then happened as a result of immigration from increasing pioneer species seed dispersal. As such, the dynamics of the two tropical forests studied are influenced by infrequent events, and the parameters *I* and *R* can contribute to detection of the relative importance of immigration and local birth processes among places or years.

Validation of the test indicated that the non‐zero‐sum *N*_sp_ test rejected neutrality properly, or more conservatively, especially when the observed *N*_sp_ was expected to be small. When real data were applied to the non‐zero‐sum *N*_sp_ test, deviations from neutrality were detected in datasets (Etienne, 2007; Jabot & Chave, 2011). The performance of our test improved when sufficient *N*_sp_ was observed, that is, when there was a large interval between the two censuses. This finding indicates that longer‐term observations can help us understand the ecological processes driving community dynamics, or at least find deviations from neutral settings (Leroi et al., 2020). Thus, census frequency and length should be considered when designing field monitoring.

We also examined the non‐zero‐sum *N*_sp_ test based on snapshot data, which is commonly used to census biological communities (see Supplementary materials). Because snapshot data cannot distinguish individuals, that is, which individuals died, survived, or were recruited between the first and second census, the definition of *N*_sp_ was also different; *N*_sp_ was defined as the number of species in which individuals were seen in the second census but not in the first (Figure S2). Simulation is still required for *d* and *r*, for which two snapshot censuses will not provide sufficient information; these parameters should be estimated or obtained from other sources. Using the snapshot data, the non‐zero‐sum *N*_sp_ test failed to reject neutrality in BCI or in Pasoh, except in one case (Table S1). The power of the test using snapshot data was apparently reduced compared with the results using individual‐based monitoring data. This finding also emphasizes the importance of long‐term individual‐based monitoring.

Although we recognize that failure to reject the non‐zero‐sum neutral model does not necessarily indicate that the community is “neutral,” conventional methods such as the SAD‐based approach could not distinguish between the processes because of the poor performance of previous neutrality tests. Rejecting neutrality is an important first step in exploring the non‐neutral processes underlying natural communities. The results of this study verified our initial hypothesis that using multiple datasets could improve the performance of neutrality tests. Although this test is useful for rejecting “neutrality” of a community, alternative models are still needed to understand the processes underlying community dynamics. Applications of the non‐zero‐sum model by adding some deterministic factors could enhance the ability to reveal factors that shape the natural community and biodiversity in future theoretical and empirical studies. Moreover, this model could help us to understand the importance of the neutrality assumption in stochastic dynamics, which is key to better predictions and management. For example, since a tropical forest remains under threat of deforestation and climate change, the effects of these drivers on forest biodiversity should be evaluated. The model can be applied to biodiversity projections under specific scenarios. Thus, the sensitivity of local/metacommunity structure and parameters to such threats may be revealed, and the key processes (birth, death, and immigration) to conserve can be identified.

## CONFLICT OF INTEREST

The authors declare no conflict of interest.

## AUTHOR CONTRIBUTIONS

**Yayoi Takeuchi**: Conceptualization (lead); formal analysis (lead); investigation (lead); methodology (lead); visualization (lead); writing—original draft (lead); writing—review & editing (lead). **Hisashi Ohtsuki**: Methodology (equal); visualization (equal); writing—original draft (supporting); writing—review & editing (supporting). **Hideki Innan:** Conceptualization (equal); methodology (equal); writing—original draft (supporting); writing—review & editing (supporting).

## Supporting information

Fig S1

Fig S2

Table S1

## Data Availability

Simulation code in C language for *N*_sp_ distribution is archived in the Dryad depository: https://doi.org/10.5061/dryad.kh189325z. Pasoh and Barro Colorado Island datasets are available through ForestGEO data request portal http://ctfs.si.edu/datarequest/.
