# Supplementary material for: Non‐zero‐sum neutrality test for the tropical rain forest community using long‐term between‐census data
Source: Ecol Evol. 2022 Jan 17;12(1):e8462. doi: 10.1002/ece3.8462 (PMC8809451; doi:10.1002/ece3.8462)
Supplement: Supplementary file 1 — Fig S1 [file ECE3-12-e8462-s002.pdf]

(a)

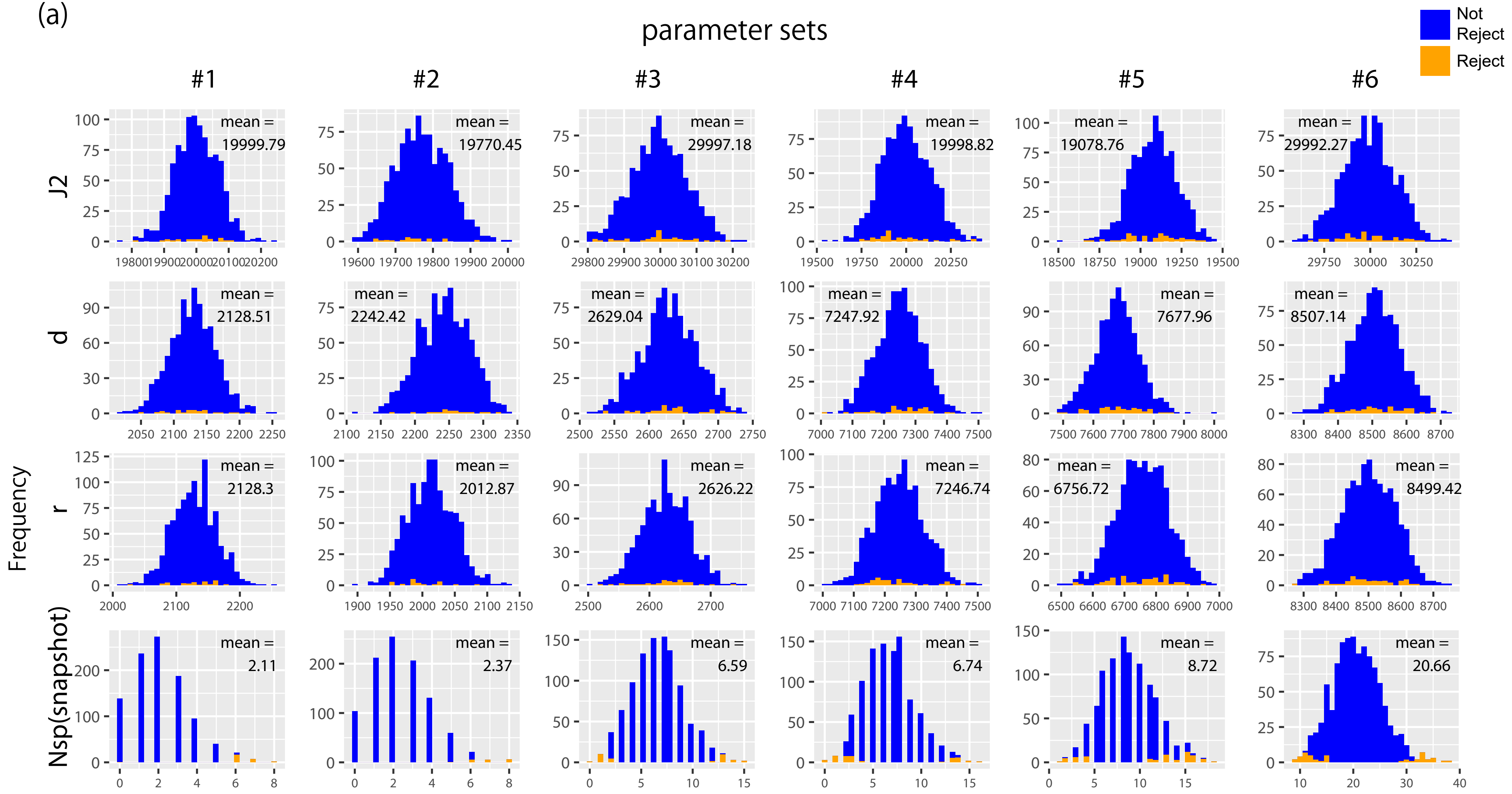

Fig. S1 (a) Histograms of the parameter values which were obtained 1000 simulations per parameter sets. The rejected cases by *Nsp* test (both-sides) were shown in orange.

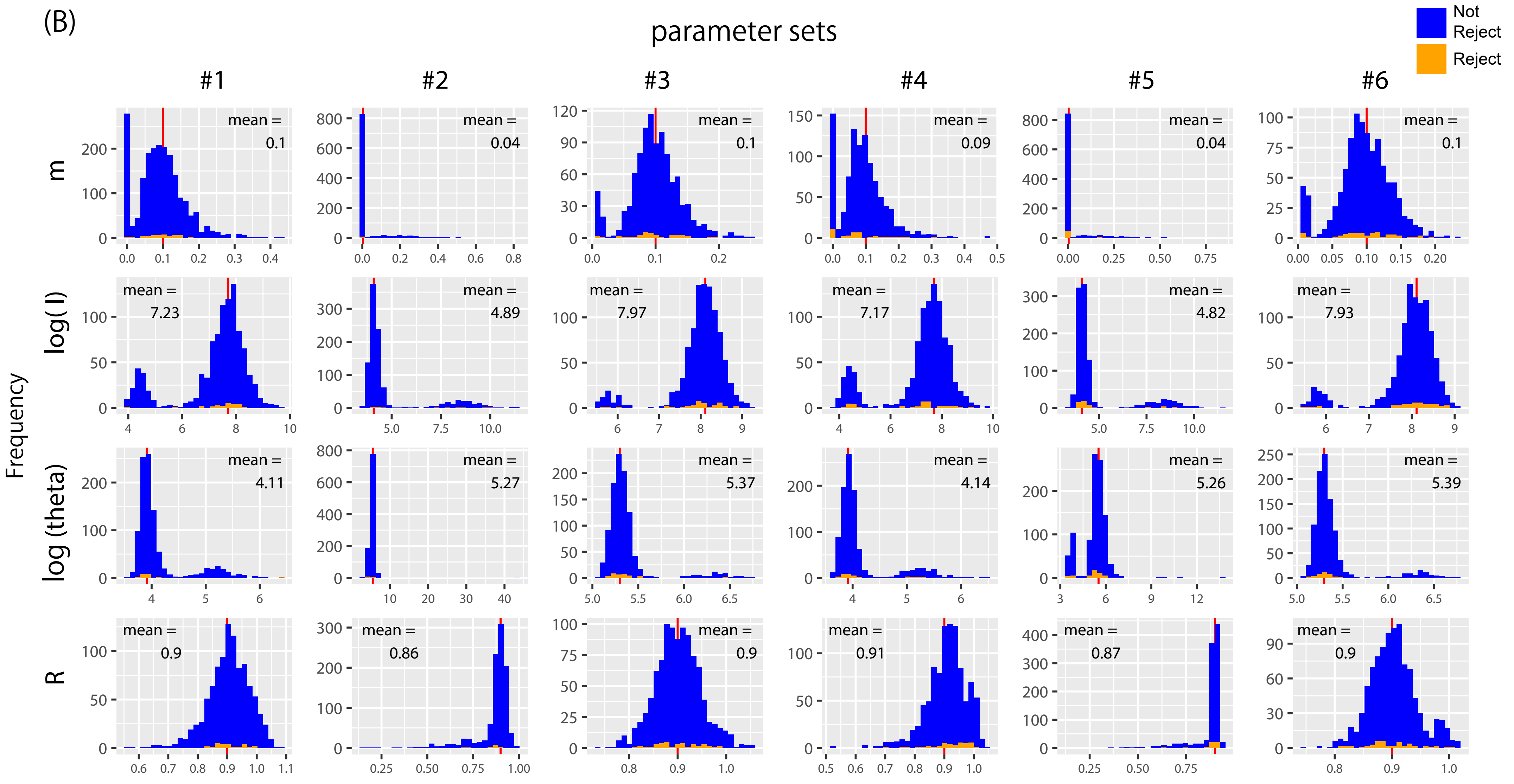

Fig. S1 (B) Frequency of the estimated parameters which were obtained for 1000 simulations per parameter sets. The rejected cases by Nsp test (two-tailed) were shown in orange.
