# Supplementary material for: Non‐zero‐sum neutrality test for the tropical rain forest community using long‐term between‐census data
Source: Ecol Evol. 2022 Jan 17;12(1):e8462. doi: 10.1002/ece3.8462 (PMC8809451; doi:10.1002/ece3.8462)
Supplement: Supplementary file 2 — Fig S2 [file ECE3-12-e8462-s001.pdf]

For snapshot data

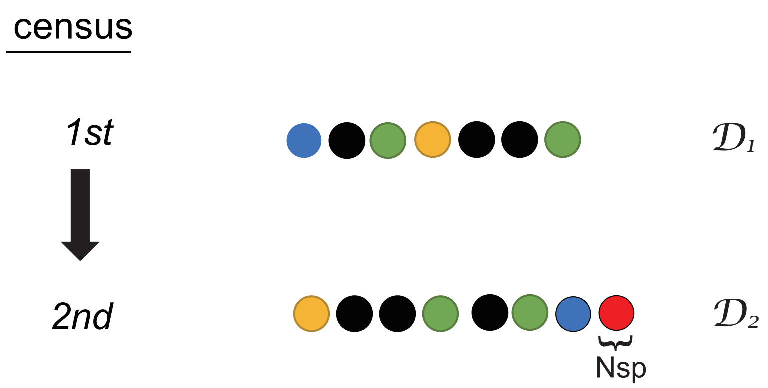

Fig. S2 Overviews of non-zerosum dynamics in a local community of snapshot data set. Here Nsp is defined as the number of the species which are seen among individuals at 2nd census but are not seen among individuals at 1st census. Although two snapshot censuses will not provide the information of d and r , you need to obtain or estimate from other sources for simulation.
